# Optimized ensemble learning framework for neonatal asphyxia prediction using perinatal clinical features

**DOI:** 10.3389/fpubh.2026.1899811

**Published:** 2026-07-29

**Authors:** Muhammad Afzal, Madiha Amjad, Saleem Ullah, Rahman Shafique, Farhan Amin, Isabel de la Torre, Atenea Ruigómez Noriega, David García Obeso

**Affiliations:** 1 Institute of Computing, Khwaja Fareed University of Engineering and Information Technology, Rahim Yar Khan, Pakistan; 2 School of Computer Science and Engineering, Yeungnam University, Gyeongsan, Republic of Korea; 3 Department of Signal Theory and Communications, University of Valladolid, Valladolid, Spain; 4 Universidad Europea del Atlántico Isabel Torres, Santander, Spain

**Keywords:** healthcare, machine learning, mother and child care, public health, risk perceptions

## Abstract

**Background:**

Neonatal asphyxia is a life-threatening perinatal condition associated with neonatal mortality and long-term neurological impairment. Therefore, early identification of high-risk newborns is essential for timely clinical intervention; however, accurate prediction of neonatal asphyxia remains a key challenge due to heterogeneous maternal and perinatal risk factors, for example, overlapping clinical patterns, and class imbalance in medical datasets. Thus, to solve this issue, this research proposes an imbalance-sensitive machine-learning framework for the prediction of neonatal asphyxia using perinatal and maternal clinical variables. In contrast to conventional SMOTE, which interpolates minority samples without explicitly prioritizing their diagnostic difficulty, the proposed framework is designed to strengthen the representation of informative asphyxia cases located in uncertain or overlapping decision regions while reducing the influence of less representative synthetic generation.

**Methods:**

In summary, in this research, a novel synthetic oversampling framework, named HEM-SMOTE, is proposed to improve minority-class representation by generating more informative synthetic asphyxia samples through hybrid distance-guided neighbor selection. The proposed method integrates local Euclidean-distance-based similarity with Mahalanobis-distance-based covariance awareness to identify representative minority neighbors prior to the generation of synthetic samples.

**Results:**

To measure the performance, we compared our proposed framework with the traditional classifiers, for instance, logistic regression, support vector machine, random forest, balanced random forest, Extra Trees, gradient boosting, XGBoost, LightGBM, and CatBoost. The comparison is performed based on accuracy, balanced accuracy, precision, recall, specificity, F1-score, and AUROC. The experimental results show that the proposed framework achieved the strongest overall performance, and achieved 95.35% accuracy as compared with conventional SMOTE and class-weighted baselines. The proposed approach provided more balanced predictive performance across discrimination and classification metrics.

## 1 Introduction

Neonatal asphyxia, also referred to as birth asphyxia, is a critical clinical condition caused by inadequate oxygen supply to a newborn during the perinatal period (1). It remains one of the major contributors to neonatal mortality and long-term neurological impairment worldwide, particularly in low- and middle-income countries (2, 3). Global health reports identify neonatal asphyxia as a leading cause of early infant death and associate it with severe complications, including cerebral palsy, developmental delay, and cognitive impairment (4, 5). Early recognition of neonates at risk of asphyxia is clinically important because delayed intervention can lead to severe and potentially irreversible outcomes (6). However, predicting neonatal asphyxia remains difficult because the condition is influenced by several interrelated maternal, obstetric, and perinatal factors (7). In routine clinical practice, risk assessment is commonly supported by expert judgment, Apgar-based evaluation, and conventional statistical approaches (8). These methods are useful for immediate clinical interpretation, but they are often limited when multiple risk factors interact in nonlinear and overlapping ways (9). Machine-learning methods have therefore been explored for neonatal outcome prediction because they can learn complex patterns from structured healthcare data (10). Previous studies have applied models such as logistic regression, support vector machines, random forests, and gradient-boosting algorithms to predict neonatal complications, including birth asphyxia (11, 12). Despite these advances, reliable clinical use remains challenging. Advanced models may behave as black-box systems, while electronic health records often contain noisy, incomplete, and unevenly distributed observations (13). In addition, high-risk neonatal cases are usually fewer than normal cases, which can bias model learning toward the majority class and reduce sensitivity for clinically critical cases (14, 15).

Recent ensemble-based approaches have shown promise because they combine the strengths of multiple learners and can better represent heterogeneous clinical variables (16–19). Nevertheless, stronger classifiers alone do not fully solve the core difficulty of neonatal asphyxia prediction. Perinatal risk factors such as fetal distress, meconium aspiration, prolonged labor, placental complications, and maternal comorbidities may appear in both affected and unaffected neonates, producing overlapping decision regions rather than clearly separable classes. This overlap highlights the need for an imbalance-aware and robust predictive framework that can improve minority-class recognition while preserving clinically meaningful patterns. Therefore, early identification of neonates at high risk of asphyxia is essential to enable timely clinical intervention and reduce the likelihood of irreversible outcomes (6).

However, neonatal asphyxia is a multifactorial condition, making its prediction clinically challenging. Its risk factors include maternal health conditions, obstetric complications, and perinatal clinical indicators, which collectively increase the uncertainty and complexity of the prediction process (7). Conventional approaches primarily rely on expert judgment, rule-based scoring systems such as the Apgar score, and basic statistical methods (8). Although these methods provide useful clinical insights, but they may not adequately capture complex nonlinear interactions among multiple perinatal risk factors, thereby limiting their predictive performance in real-world clinical scenarios (9). In recent years, machine learning (ML) methods have garnered increasing attention in medical diagnosis and prognosis due to their ability to model complex relationships within healthcare data (10). Several studies have investigated the use of ML classifiers, for instance, logistic regression (LR), support vector machines (SVM), random forests (RF), and gradient boosting algorithms (GBM), for the prediction of neonatal complications such as asphyxia (11, 12). However, the clinical deployment of these models remains challenging due to the black-box nature of advanced algorithms and the presence of noise in electronic health records (EHRs) (13). Although these approaches have demonstrated promising results, the performance of individual predictive models is often limited when applied to imbalanced and noisy medical datasets, where high-risk cases may constitute only a relatively small proportion of the overall sample (14, 15). Recent studies have increasingly adopted machine-learning and ensemble-based models for neonatal risk prediction because these methods can capture nonlinear relationships among heterogeneous clinical and perinatal variables (16–19). However, the improvement obtained from stronger classifiers alone does not fully address the central difficulty in neonatal asphyxia prediction. Perinatal datasets often contain partially overlapping risk patterns between asphyxiated and non-asphyxiated neonates, where predictors such as fetal distress, meconium aspiration, prolonged labor, placental complications, and maternal conditions may not form clearly separable decision regions. In addition, the minority class represents clinically critical asphyxia cases, making false-negative predictions more harmful than false positives. As a result, models may report acceptable overall accuracy while still failing to identify high-risk neonates with sufficient sensitivity. Therefore, the unresolved challenge is not only to improve classification accuracy, but also to improve the learning representation of difficult and clinically important minority-class cases (20–23) in the presence of overlapping perinatal risk factors, feature redundancy, and asymmetric clinical consequences. To address this gap, this study proposes an ML framework for neonatal asphyxia prediction. In contrast to conventional SMOTE, which interpolates minority samples without explicitly prioritizing their diagnostic difficulty, the proposed framework is designed to strengthen the representation of informative asphyxia cases located in uncertain or overlapping decision regions while reducing the influence of less representative synthetic generation. The proposed approach aims to provide a more reliable and interpretable framework for early prediction of risk of asphyxia in newborns, particularly in settings where timely recognition of high-risk newborns is essential (24–26). The key contributions of this research are summarized as follows:

- A optimized ensemble learning Framework is proposed using engineered perinatal risk factors, where the complete feature space is first examined to establish baseline predictive behavior and then refined into a compact set of informative predictors to reduce redundancy, improve interpretability, and support more reliable minority-class learning.
- A imbalance-aware experimental framework is proposed to separate the effects of feature refinement, algorithm-level cost adjustment, data-level oversampling, and model-level generalization by systematically comparing class-weighted learning, conventional SMOTE, and stacking/ensemble strategies under a consistent selected-feature setting.
- A hard-example-guided synthetic oversampling strategy, HEM-SMOTE, is proposed to improve neonatal asphyxia detection by prioritizing difficult minority samples located in overlapping or uncertain perinatal risk regions, enabling more informative synthetic sample generation than conventional SMOTE and enhancing clinically important asphyxia-class prediction.

## 2 Related work

This section presents a review of existing work on neonatal asphyxia prediction, including traditional clinical assessment methods, statistical models, machine learning approaches, and strategies for handling imbalances.

### 2.1 Traditional state-of-the-art methodologies

Conventional assessment of neonatal asphyxia has largely relied on clinical grading scales and statistical analysis of perinatal variables. The Apgar score, introduced in 1953, remains one of the most widely used clinical tools for evaluating neonatal well-being shortly after birth (8). However, previous studies have shown that Apgar scoring can be influenced by maternal sedation, gestational age, and inter-observer variability, making it subjective and limited in predicting long-term neurological outcomes (27). Conventional statistical models, including multivariate logistic regression, have also been used to identify risk factors such as prolonged labor and meconium-stained liquor (6, 7). Although these methods provide useful clinical interpretation, they are primarily based on linear assumptions and may not adequately capture the high-dimensional and nonlinear relationships present in complex perinatal data (28).

### 2.2 Machine learning for neonatal prediction

With the increasing digitalization of healthcare, ML has emerged as an effective tool for neonatal risk stratification. Early ML-based studies for birth-related distress prediction employed algorithms such as SVM and k-nearest neighbors (KNN) (29). These methods demonstrated improved predictive capability compared with traditional regression-based approaches; however, their performance can be limited when handling high-dimensional EHR data. More recent studies have reported that tree-based models, such as random forests and standard gradient boosting algorithms, are more effective in modeling nonlinear clinical relationships (11, 30). However, single-model approaches often remain vulnerable to noisy medical datasets commonly encountered in real-world clinical environments, where input noise, irrelevant predictors, or weak feature representations may contribute to overfitting and reduced generalization (10, 13).

### 2.3 Ensemble learning in medical diagnosis

Ensemble learning has gained considerable attention in medical diagnosis because it combines the strengths of multiple classifiers and reduces the limitations associated with individual models (15). In neonatal care, bagging and boosting techniques have been applied to predict conditions such as preterm birth and neonatal sepsis (18, 22). In particular, stacking-based ensemble approaches, in which a meta-learner integrates the outputs of multiple base learners, have shown improved classification performance compared with simple majority-voting strategies in several medical prediction tasks (12, 20). Recent studies have also explored advanced boosting models such as XGBoost and LightGBM for tabular healthcare data, showing their effectiveness in handling heterogeneous features and missing values (16, 17). However, many ensemble-based studies still focus primarily on improving overall accuracy, which may not be sufficient in clinical applications where minority-class detection is more important than global performance (22).

### 2.4 Limitations and research gap

Despite the progress achieved through clinical scoring systems, statistical models, machine-learning classifiers, and ensemble-based approaches, several methodological limitations remain unresolved in neonatal asphyxia prediction. Most existing studies are predominantly model-centric, with emphasis placed on classifier selection, algorithmic complexity, and hyperparameter optimization, while comparatively limited attention is given to the statistical quality of the training data, feature redundancy, and class-distribution effects (14, 19, 23). This is an important limitation because neonatal asphyxia datasets commonly contain heterogeneous and partially overlapping perinatal risk factors, where asphyxiated and non-asphyxiated neonates may share similar clinical profiles. In such settings, improving the classifier alone may not be sufficient to produce reliable discrimination, particularly for high-risk minority cases.

Another important limitation is that performance is often reported using overall accuracy or ROC-AUC, which may overestimate clinical usefulness when the minority class represents asphyxiated neonates. Since false-negative predictions may delay critical intervention, neonatal asphyxia prediction requires stronger attention to sensitivity, F1-score, balanced accuracy, and precision–recall-based evaluation rather than accuracy alone (20–22). Although conventional imbalance-handling techniques such as class weighting and SMOTE have been used in medical prediction tasks, these methods do not explicitly focus on difficult or borderline minority cases located in overlapping decision regions. As a result, synthetic samples may be generated from less informative regions of the minority class, limiting their ability to strengthen clinically meaningful asphyxia detection. These gaps motivate the proposed HEM-SMOTE framework, which shifts the focus from purely model-centric optimization toward hard-example-guided minority oversampling. By improving the representation of informative asphyxia cases before model training, the proposed framework aims to enhance minority-class learning and provide a more clinically meaningful prediction strategy for early neonatal asphyxia detection. Table 1 presents a concise overview of recent studies.

**Table 1:** Summary of recent machine-learning studies on neonatal asphyxia and related neonatal clinical outcomes.

Ref.	Classifiers	Feature Eng.	Data balancing	Accuracy	Key limitation
Sheikhtaheri et al. (41)	SVM, RF, ANN, C5.0, CHAID, BN, Ensemble	Yes	NR	0.98 AUC by SVM/Ensemble	Predicted neonatal mortality rather than direct asphyxia occurrence.
Darsareh et al. (34)	RF and comparative classifiers	Yes	NR	0.99 by RF	Class imbalance and false-negative reduction were not explicitly handled.
Ribeiro et al. (42)	BLR with CTG-derived features	Yes	NR	94.93% AUC by BLR-CR2	Very small pathological class limited minority-class generalization.
Robi and Sitote (40)	Stacking model	NR	NR	97.04% by Stacking	Birth asphyxia was treated as one class in a multi-disease setting.
Uddin et al. (43)	LR + KNN Ensemble	Yes	ROS	99.29% by LR + KNN	Used infant-cry features rather than routine perinatal clinical predictors.
Dey et al. (35)	LR, ANN, Hybrid CNN	Yes	ROS	99.16% by LR	ROS may increase duplication-based overfitting.
Alhasson et al. (38)	RF	Yes	NR	96% by RF	Predicted low APGAR score rather than direct asphyxia diagnosis.
Malakooti et al. (44)	RF and comparative classifiers	Yes	NR	0.87 by RF	Target was NICU admission rather than neonatal asphyxia.
Keles et al. (45)	XGBoost	NR	NR	0.95 AUC by XGBoost	Predicted AKI/survival after neonatal encephalopathy.
Zhang et al. (46)	NB with RFECV	Yes	SMOTE	0.923 AUC by NB	Target was AKI in asphyxiated neonates, not primary asphyxia prediction.
Al Mashrafi et al. (47)	LR, LDA, RF	Yes	SMOTE, Cost-sensitive	AUC-PR > 0.85 by LR/LDA/RF	Focused on neonatal mortality rather than asphyxia detection.

ANN, artificial neural network; BN, Bayesian network; CHAID, chi-square automatic interaction detection; BLR, binary logistic regression; CTG, cardiotocography; CR2, compression ratio scale 2; CNN, convolutional neural network; NB, naive Bayes; RFECV, recursive feature elimination with cross-validation; LDA, linear discriminant analysis; AKI, acute kidney injury; ROS, random oversampling; SMOTE, synthetic minority oversampling technique; NR, not reported.

## 3 Proposed framework

**Figure 1:**
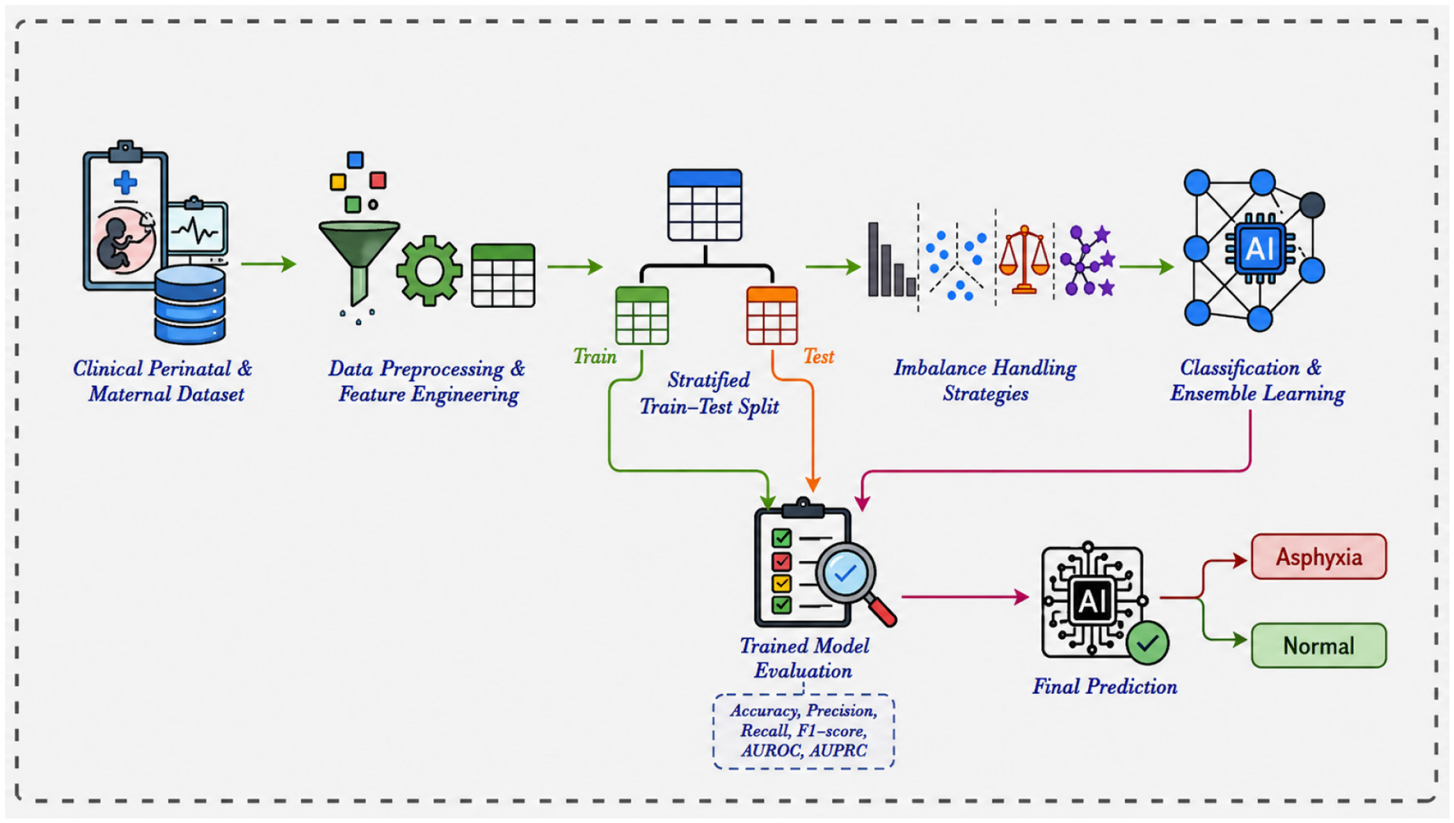
Proposed framework.

Figure 1 illustrates the proposed framework for neonatal asphyxia prediction. The framework follows a controlled machine-learning pipeline beginning with a structured clinical dataset containing maternal and perinatal risk factors. These include maternal age, hypertension, diabetes, placental complications, premature rupture of membranes, meconium aspiration, fetal distress, multiple gestation, and prolonged labor. Before model development, records with missing target labels are removed, non-predictive identifiers such as patient ID are excluded, and categorical variables are encoded where required to obtain a machine-learning-ready feature matrix. The prepared dataset is then divided into training and testing subsets using a stratified train-test split. This strategy preserves the original distribution of asphyxia and normal cases in both subsets and supports unbiased model evaluation. To prevent information leakage, imbalance-handling techniques are applied only to the training data, while the held-out test set remains unchanged for final evaluation.

After data preparation, several imbalance-aware learning conditions are investigated. The first condition uses the original training data as a baseline without any class-balancing strategy. The second applies standard SMOTE as a conventional oversampling method. The third uses class-weighted learning, where higher misclassification penalties are assigned to the minority asphyxia class. The fourth condition applies the proposed HEM-SMOTE method, which generates synthetic minority samples using a hybrid distance-based neighbor selection strategy. Unlike conventional SMOTE, HEM-SMOTE combines Euclidean and Mahalanobis distance measures during minority-neighbor selection. Euclidean distance captures direct local similarity between samples, whereas Mahalanobis distance incorporates covariance-aware relationships among clinical risk factors. By combining these distance measures, HEM-SMOTE aims to generate more informative synthetic asphyxia samples from clinically relevant minority regions. Following imbalance handling, multiple machine-learning models are trained and compared, including LR, SVM, RF, Balanced RF, Extra Trees, Gradient Boosting, XGBoost, LightGBM, and CatBoost. Ensemble strategies, including stacking and voting, are also evaluated to examine whether combined learners improve predictive generalization over individual classifiers. In the stacking configuration, heterogeneous base learners generate probability outputs, which are then integrated by a meta-learner to produce the final prediction. Model performance is assessed using accuracy, balanced accuracy, precision, recall/sensitivity, specificity, F1-score, AUROC, AUPRC, and confusion-matrix analysis. These metrics are used because overall accuracy alone may be misleading in neonatal asphyxia prediction, where failure to detect asphyxiated neonates can have serious clinical consequences. The final prediction stage classifies each case as either asphyxia or normal based on the trained and evaluated model.

### 3.1 Data source and collection process

The main dataset in this research was obtained from Teaching Hospital Dera Ghazi Khan (DHQ), Punjab, Pakistan. The sample comprises 20,520 perinatal and maternal clinical records from routine obstetric care between 2023 and 2025. All records were collected with written informed consent from the participants or their legal guardians. The study protocol was reviewed by the Institutional Review Board (IRB) of The Islamia University of Bahawalpur, and all procedures were conducted in accordance with the ethical principles of the Declaration of Helsinki (31). The dataset was collected to predict neonatal asphyxia, a serious outcome associated with poor short-term and long-term health effects. The records correspond to individual delivery events and include maternal health indicators, obstetric complications, labor-related conditions, and fetal risk factors recorded during the perinatal period. The prediction task is formulated as a binary classification problem, where the target variable *y* is defined as in ref of Equation 1:


$$y=\{\begin{array}{ll} 1, & \text{if infant asphyxia is present}\\ 0, & \text{if infant asphyxia is absent} \end{array}$$ (1)

### 3.2 Dataset description

The dataset contains structured maternal, obstetric, and fetal/perinatal clinical variables collected during routine obstetric care. These features represent clinically relevant risk factors associated with neonatal asphyxia. The maternal and obstetric variables describe the mother's demographic and pregnancy-related conditions, whereas the fetal/perinatal variables describe delivery-related and newborn-associated complications. Furthermore, the target variable is asphyxia. The detailed description of the dataset variables is presented in Table 2.

**Table 2:** Description of dataset features.

Variable	Description
Maternal features	
MA	Maternal age
MH	Maternal hypertension
MD	Maternal diabetes
MG	Multiple gestation
PL	Prolonged labor
PA	Placental abruption
Child/perinatal features	
PP	Placental previa
PM	Premature rupture of membranes
MEC	Meconium aspiration
FD	Fetal distress

A sample of the structured dataset used in this study is presented in Table 3. The table shows representative patient-level records after clinical variables were organized into a tabular format. Each row corresponds to an individual delivery case, while each column represents a maternal or perinatal feature used for neonatal asphyxia prediction. The target column indicates the asphyxia outcome, which was originally recorded as a categorical label, where “YES” represents the presence of neonatal asphyxia and “NO” represents its absence. For machine-learning implementation, these labels were encoded into binary numerical values, with 1 indicating asphyxia-positive cases and 0 indicating asphyxia-negative cases.

**Table 3:** Sample records from the dataset.

MA	MH	MD	PA	PP	PM	MEC	FD	MG	PL	Asphyxia
20	Yes	No	No	Yes	No	No	No	No	No	Yes
25	No	Yes	No	Yes	Yes	Yes	No	Yes	No	No
24	No	Yes	No	Yes	No	No	Yes	No	No	No
20	No	No	No	Yes	No	No	No	No	No	No
30	Yes	No	Yes	No	No	Yes	Yes	Yes	Yes	Yes
30	No	No	No	Yes	No	Yes	No	Yes	Yes	Yes
25	Yes	Yes	No	Yes	No	Yes	No	Yes	No	No
25	No	No	No	Yes	No	No	Yes	Yes	Yes	No
30	No	No	No	Yes	No	No	No	Yes	Yes	No
30	No	No	No	Yes	No	No	No	Yes	No	No

**Figure 2:**
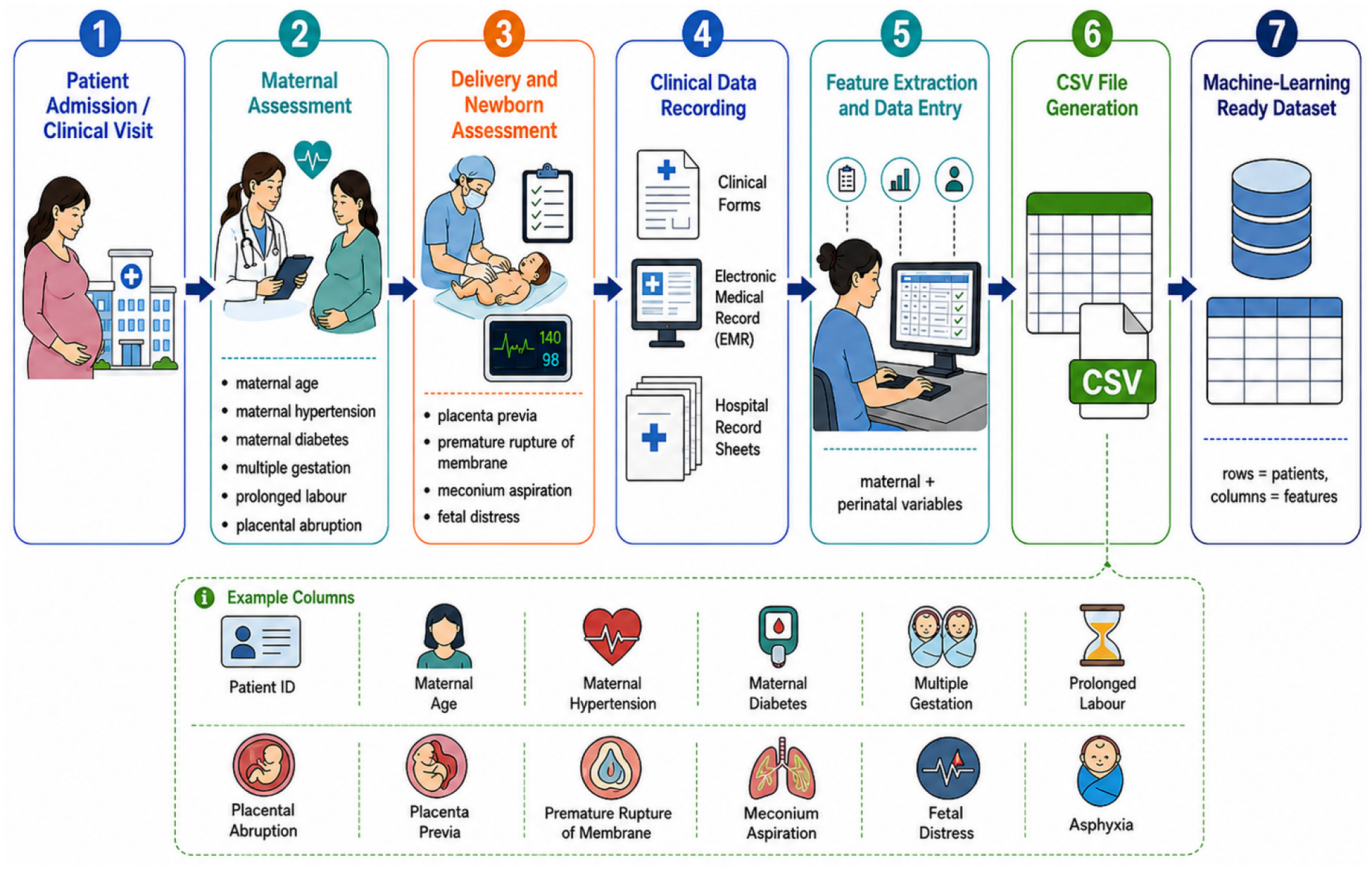
Data collection and CSV preparation.

The process through which these patient-level samples were obtained and converted into a machine-learning-compatible dataset is illustrated in Figure 2. The workflow begins with patient admission and clinical assessment of the mother, followed by delivery and newborn evaluation. Maternal and perinatal information is then recorded from clinical forms, electronic medical records, and hospital record sheets. After this stage, relevant variables are extracted, entered into a structured format, and exported as a CSV file. Finally, the CSV file is converted into a machine-learning-ready dataset, where rows represent patient records and columns represent predictive clinical features.

### 3.3 Data preprocessing

Data preprocessing is necessary in tabular medical data to handle missing, inconsistent, and noisy values, ensuring the dataset is accurate and reliable for analysis (32). It improves data quality and enhances the performance and generalization of machine learning models for better clinical decision-making. Although the dataset was collected carefully, the possibility of missing or incomplete values could not be ignored. Therefore, records containing missing values in the target variable or any input feature were removed from the dataset. Categorical variables were transformed into numerical form using label encoding, where binary responses such as “YES” and “NO” were encoded as 1 and 0, respectively. In addition, feature scaling was applied using standardization to reduce the effect of different numerical ranges among input variables and to ensure consistent model training.

### 3.4 Data split

The cleaned dataset was divided into training and testing subsets using a stratified 80:20 split. In this setting, 80% of the samples were used for model training, whereas the remaining 20% were reserved for independent testing. Stratification (33) was applied during the split to preserve the original class distribution of asphyxia-positive and asphyxia-negative cases in both subsets. This step is important because the dataset contains a moderate class imbalance, and a random split may produce unequal class proportions, which can bias model training and evaluation. By maintaining similar class ratios in the training and testing sets, the models can be trained on a representative distribution and evaluated more reliably on unseen data. The class-wise distribution of the training and testing subsets is presented in Table 4.

**Table 4:** Training and testing data distribution.

Class	Training	Testing	Total
1	6,960	1,740	8,700
0	9,456	2,364	11,820
**Total**	**16,416**	**4,104**	**20,520**

Bold values indicate the aggregate totals across both classes for the training, testing, and complete datasets.

### 3.5 Feature engineering

**Figure 3:**
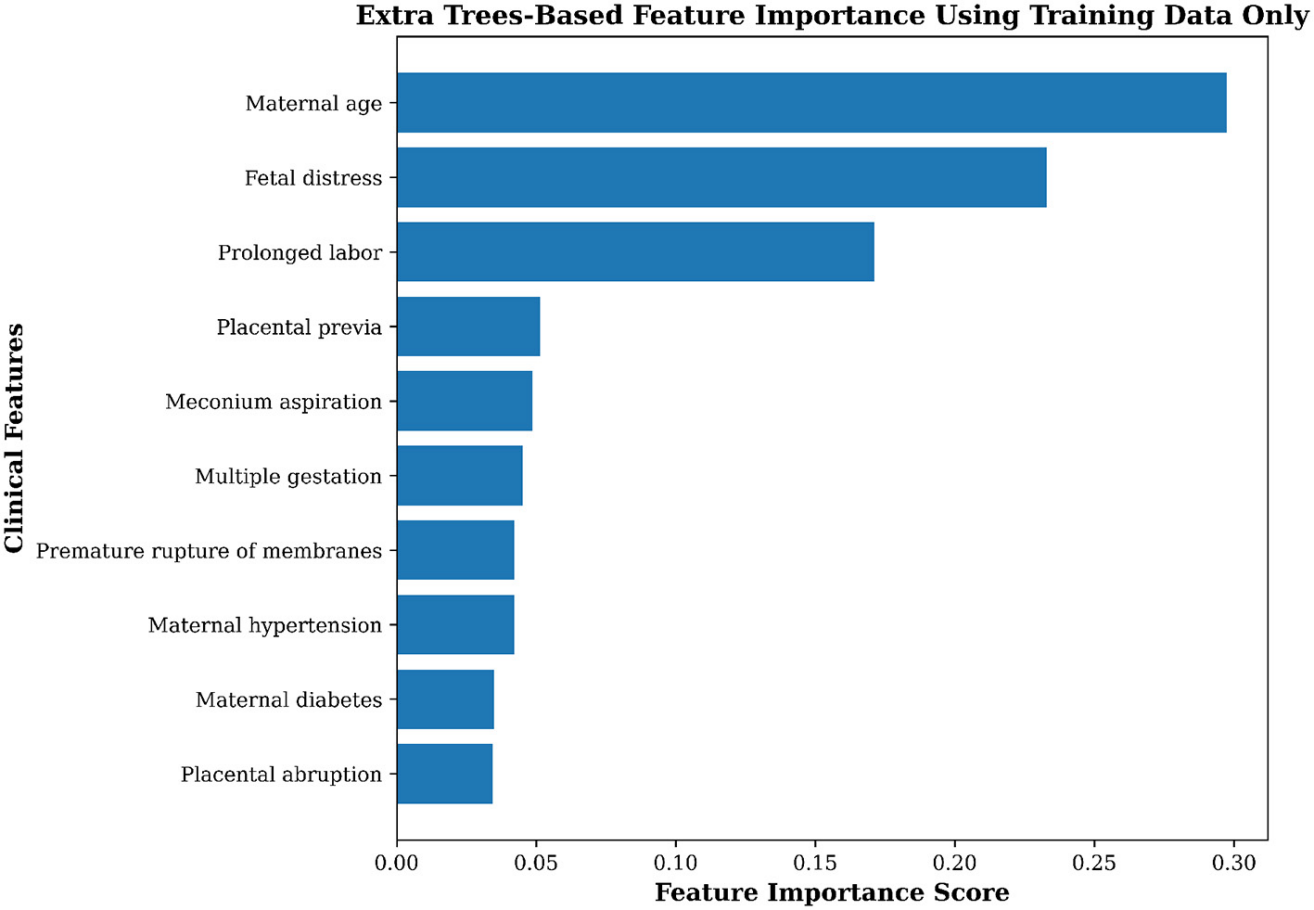
Extra Trees-based feature importance scores.

After data preprocessing, the final feature matrix consisted of 10 clinically relevant maternal and perinatal variables. These variables represent risk factors commonly assessed during obstetric and neonatal care. Maternal age was retained as a continuous numerical feature, whereas the remaining variables were encoded as binary clinical indicators representing the presence or absence of specific maternal, placental, labor-related, or fetal complications. To examine the predictive contribution of each variable, feature importance analysis was performed using an Extra Trees classifier. This analysis was used to assess the relative influence of the input features with respect to the neonatal asphyxia outcome. As illustrated in Figure 3, maternal age, fetal distress, and prolonged labor showed comparatively higher importance scores than the remaining variables, indicating their stronger contribution to model discrimination.

Although the importance scores varied across features, all variables demonstrated predictive relevance and were therefore retained for model development. No feature was removed at this stage because the selected variables are clinically meaningful and collectively represent different dimensions of neonatal asphyxia risk, including maternal health status, placental complications, labor-related conditions, and fetal distress indicators.

### 3.6 Proposed framework

**Figure 4:**
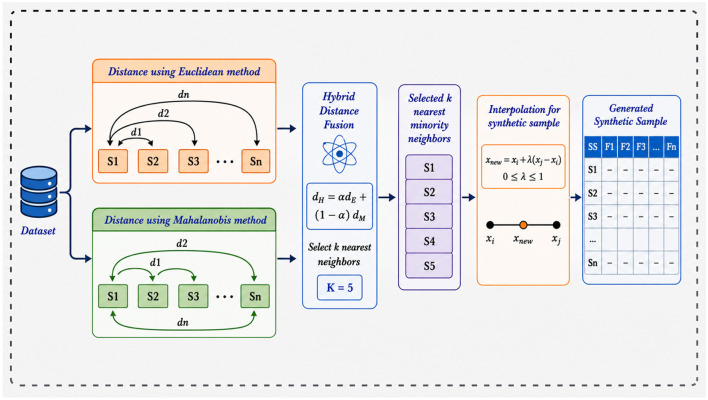
Architecture of the proposed framework.

To address class imbalance in neonatal asphyxia prediction, this study proposes a hybrid oversampling method termed the Hybrid Euclidean–Mahalanobis Synthetic Minority Oversampling Technique (HEM-SMOTE). The internal workflow of the proposed HEM-SMOTE method is illustrated in Figure 4. The objective of this method is to generate synthetic minority-class samples by selecting more representative minority neighbors through a hybrid distance mechanism. The process begins with the minority dataset *S*, which contains samples belonging to the minority class. Let the minority-class sample set be represented as in ref of Equation 2


$$\begin{array}{l} S_{\min}=\left\{x_{1},x_{2},x_{3},\ldots ,x_{n}\right\}, \end{array}$$ (2)

where each sample *x*_*i*_ contains *p* clinical features, such as maternal age, hypertension, diabetes, placental conditions, fetal distress, and labor-related factors.

For each selected minority sample *x*_*i*_, two types of distances are calculated with respect to the other minority samples. The first branch computes the Euclidean distance, which measures the direct geometric distance between two samples. It is defined as


$$\begin{array}{l} d_{E}\left(x_{i},x_{j}\right)=\sqrt{\overset{p}{\underset{k=1}{\sum }}{\left(x_{ik}-x_{jk}\right)}^{2}}, \end{array}$$ (3)

where *x*_*i*_ is the selected minority sample, *x*_*j*_ is another minority sample, and *p* is the number of features.

The second branch computes the Mahalanobis distance. This distance considers the covariance structure among features and is useful when clinical variables are correlated. It is calculated as in ref of Equation 4:


$$\begin{array}{l} d_{M}\left(x_{i},x_{j}\right)=\sqrt{{\left(x_{i}-x_{j}\right)}^{T}\Sigma^{-1}\left(x_{i}-x_{j}\right)}, \end{array}$$ (4)

where Σ^−1^ is the inverse covariance matrix of the minority-class samples.

After computing both distances, HEM-SMOTE combines them using a hybrid distance score. The hybrid distance is defined as in ref of Equation 5:


$$\begin{array}{l} d_{H}=\alpha d_{E}+\left(1-\alpha \right)d_{M},\;0\leq \alpha \leq 1, \end{array}$$ (5)

where *d*_*H*_ is the final hybrid distance, *d*_*E*_ is the Euclidean distance, *d*_*M*_ is the Mahalanobis distance, and α controls the contribution of each distance. When α = 0.5, both Euclidean and Mahalanobis distances contribute equally. Using the hybrid distance *d*_*H*_, the method selects the *k* nearest minority neighbors. In this study, *k* = 5 is used. These selected neighbors represent the most suitable minority samples for generating new synthetic samples because they are close to the selected sample in both local and covariance-aware feature spaces. Once the nearest neighbors are selected, one neighbor *x*_*j*_ is randomly chosen. A new synthetic sample is then generated by interpolation between the selected sample *x*_*i*_ and the chosen neighbor *x*_*j*_ as in ref of Equation 6 follows:


$$\begin{array}{l} x_{\text{new}}=x_{i}+\lambda \left(x_{j}-x_{i}\right),\;0\leq \lambda \leq 1, \end{array}$$ (6)

where λ is a random number between 0 and 1. This equation generates a new sample between *x*_*i*_ and *x*_*j*_ in the feature space.

For example, if *x*_*i*_ and *x*_*j*_ are two minority samples, the new synthetic sample is generated feature-wise as in ref of Equation 7:


$$\begin{array}{l} x_{\text{new},k}=x_{ik}+\lambda \left(x_{jk}-x_{ik}\right),\;k=1,2,\ldots ,p. \end{array}$$ (7)

Thus, each feature value of the synthetic sample is created by moving from the selected sample toward the chosen neighboring sample. After generating the synthetic sample, the values are clipped to remain within the valid range of the original feature space. For binary features, such as the presence or absence of a clinical condition, the generated values are rounded to either 0 or 1. This step ensures that the synthetic samples remain clinically meaningful and suitable for model training. Finally, the generated synthetic sample is added to the minority class. This process is repeated until the required number of minority-class samples is generated. The final output is the HEM-SMOTE resampled dataset, which is then used to train the classification models. Algorithm 1 shows the proposed HEM-SMOTE procedure.

Algorithm 1 **Proposed algorithm.**


 1:  **Input:**
 2:  Minority class dataset with *n* features and *m* samples
 3:  Number of neighbors *k*, hybrid weight α
 4:  **Output:**
 5:  Synthetic samples
 6:  **while** required number of samples not generated **do**
 7:        Select a minority-class sample *x*_*i*_                          ⊳ Step 1
 8:        Compute Euclidean distance *d*_*E*_ between *x*_*i*_ and other samples                 ⊳ Step 2
 9:        Compute Mahalanobis distance *d*_*M*_ between *x*_*i*_ and other samples           ⊳ Step 3
10:        Compute hybrid distance:
11:                *d*_*H*_ = α*d*_*E*_ + (1 − α)*d*_*M*_                        ⊳ Step 4
12:        Select *k* nearest neighbors using *d*_*H*_                      ⊳ Step 5
13:        Randomly choose a neighbor *x*_*j*_                      ⊳ Step 6
14:        Generate a synthetic sample:
15:                *x*_new_ = *x*_*i*_ + λ(*x*_*j*_ − *x*_*i*_)                          ⊳ Step 7
16:                where 0 ≤ λ ≤ 1
17:        Clip *x*_new_ to valid feature range and adjust binary attributes              ⊳ Step 8
18:        Add *x*_new_ to the minority class                        ⊳ Step 9
19:  **end** **while**

### 3.7 ML hyperparameter settings

The hyperparameter settings as shown in Table 5 were selected to ensure a fair and reproducible comparison among the evaluated classifiers while reducing the risk of overfitting. For tree-based models, parameters such as the number of estimators, maximum depth, minimum samples per split, and minimum samples per leaf were tuned to balance predictive performance and model generalization. For boosting models, learning rate, tree depth, subsampling, and the number of estimators were adjusted to control model complexity and improve stability on the structured perinatal dataset. For linear and margin-based models, regularization and kernel-related parameters were selected to maintain robust decision boundaries. The tuning ranges were kept clinically and computationally practical, allowing each model to be optimized under comparable conditions. In addition, all hyperparameter optimization was performed using the training data only, ensuring that the held-out test set remained independent for unbiased performance evaluation.

**Table 5:** Optimized hyperparameter settings for the evaluated models.

Model/method	Hyperparameters settings	Tuning range
LR	max_iter = 3000, random_state = 42	Fixed setting; scaling applied before model training.
SVM	kernel = linear, *C* = 1.0, probability = True	kernel ∈ {linear, polynomial, RBF}; *C* ∈ {1.0, 3.0, 5.0}.
RF	n_estimators = 300, max_depth = None, min_samples_split = 2, min_samples_leaf = 1, max_features = sqrt, random_state = 42	n_estimators ∈ {300, 500}; max_depth ∈ {None, 10, 20}; min_samples_split ∈ {2, 5}; min_samples_leaf ∈ {1, 2}; max_features ∈ {sqrt, None}.
RF(w)	n_estimators = 300, max_depth = None, class-balanced sampling, random_state = 42	n_estimators ∈ {100, 300, 500}; max_depth ∈ {None, 10, 20}; class-balancing enabled.
ETC	n_estimators = 300, max_depth = None, min_samples_split = 2, min_samples_leaf = 2, max_features = sqrt, random_state = 42	n_estimators ∈ {300, 500}; max_depth ∈ {None, 10, 20}; min_samples_split ∈ {2, 5}; min_samples_leaf ∈ {1, 2}; max_features ∈ {sqrt, None}.
XGBM	n_estimators = 300, max_depth = 5, learning_rate = 0.1, subsample = 0.8, colsample_bytree = 0.9, eval_metric = logloss, random_state = 42	n_estimators ∈ {200, 300}; max_depth ∈ {4, 5, 6}; learning_rate ∈ {0.03, 0.05, 0.1}; subsample ∈ {0.8, 0.9, 1.0}; colsample_bytree ∈ {0.8, 0.9, 1.0}.
LGBM	n_estimators = 300, learning_rate = 0.1, num_leaves = 31, max_depth = 8, subsample = 0.9, random_state = 42	n_estimators ∈ {200, 300, 500}; learning_rate ∈ {0.03, 0.05, 0.1}; num_leaves ∈ {15, 31, 63}; max_depth ∈ {-1, 8, 12}; subsample ∈ {0.8, 0.9, 1.0}.
CatBoost	iterations = 300, learning_rate = 0.05, depth = 6, loss_function = Logloss, random_state = 42	iterations ∈ {200, 300, 500}; learning_rate ∈ {0.03, 0.05, 0.1}; depth ∈ {4, 6, 8}.
SMOTE	sampling_strategy = auto, k_neighbors = 5, random_state = 42	k_neighbors ∈ {3, 5, 7}; sampling_strategy ∈ {auto, 1.0}.
HEM-SMOTE	sampling_strategy = 1.0, k_neighbors = 5, hybrid distance = Euclidean–Mahalanobis, random_state = 42	k_neighbors ∈ {3, 5, 7}; sampling_strategy ∈ {0.8, 1.0}; hybrid distance weights used for minority-neighbor selection.

## 4 Simulation and results

The experimental results are presented as a progressive evaluation framework rather than as independent model comparisons. First, baseline performance is examined on the original feature-engineered dataset to understand model behavior under the natural data distribution. Subsequently, imbalance-aware strategies are evaluated on the selected perinatal feature subset to ensure a consistent and clinically meaningful comparison. Conventional SMOTE is first used to assess the effect of data-level oversampling, followed by class-weighted and imbalance-aware models to examine algorithm-level cost adjustment. Stacking, threshold tuning, and voting ensembles are then evaluated to investigate model-level generalization. Finally, the proposed HEM-SMOTE approach is assessed to determine whether hard-example-guided oversampling can improve minority-class representation and clinically important asphyxia detection.

### 4.1 Experimental setup and evaluation metrics

The proposed approach was experimentally evaluated on a Windows operating system using a Core i7 12th-generation machine. The system was equipped with 64 GB RAM and a 1 TB SSD. The implementation was carried out in Python using Jupyter Notebook. Several scientific computing and machine-learning libraries, including TensorFlow, Keras, scikit-learn, pandas, NumPy, XGBoost, LightGBM, CatBoost, and imbalanced-learn, were used for data preprocessing, model development, oversampling, training, and evaluation. To assess the predictive performance of the proposed neonatal asphyxia prediction framework, multiple evaluation metrics were used. Since the task is a binary classification problem, the confusion matrix consists of true positives (TP), true negatives (TN), false positives (FP), and false negatives (FN). In this study, TP represents correctly predicted asphyxia-positive cases, TN represents correctly predicted non-asphyxia cases, FP represents non-asphyxia cases incorrectly predicted as asphyxia, and FN represents asphyxia cases incorrectly predicted as non-asphyxia. Accuracy measures the overall proportion of correctly classified samples and is defined as in ref of Equation 8:


$$\begin{array}{l} Accuracy=\frac{TP+TN}{TP+TN+FP+FN} \end{array}$$ (8)

Precision measures the proportion of correctly predicted asphyxia-positive cases among all samples predicted as positive in ref of Equation 9:


$$\begin{array}{l} Precision=\frac{TP}{TP+FP} \end{array}$$ (9)

Recall, also known as sensitivity, measures the ability of the model to correctly identify asphyxia-positive cases as in ref of Equation 10:


$$\begin{array}{l} Recall=Sensitivity=\frac{TP}{TP+FN} \end{array}$$ (10)

Specificity measures the ability of the model to correctly identify non-asphyxia cases as in ref of Equation 11:


$$\begin{array}{l} Specificity=\frac{TN}{TN+FP} \end{array}$$ (11)

The F1-score provides a harmonic mean of precision and recall and is particularly useful when evaluating models on imbalanced datasets as in ref of Equation 12:


$$\begin{array}{l} F1\text{-}score=2\times \frac{Precision\times Recall}{Precision+Recall} \end{array}$$ (12)

Balanced accuracy was also used because the dataset contains a moderate class imbalance. It considers both sensitivity and specificity as in ref of Equation 13:


$$\begin{array}{l} Balanced\;Accuracy=\frac{Sensitivity+Specificity}{2} \end{array}$$ (13)

In addition to threshold-dependent metrics, probability-based metrics were also used. The area under the receiver operating characteristic curve (AUROC) evaluates the model's ability to distinguish between asphyxia-positive and asphyxia-negative cases across different classification thresholds. The area under the precision-recall curve (AUPRC) was also reported because it is informative for imbalanced classification tasks, particularly when the positive class is clinically important.

### 4.2 Baseline performance comparison

The first experiment was conducted on the original feature-engineered dataset without resampling, class weighting, or any imbalance-handling strategy. This experiment served as the baseline evaluation under the natural class distribution. As shown in Table 6, tree-based models achieved better performance than linear and kernel-based models, suggesting that neonatal asphyxia prediction involves nonlinear relationships and interactions among maternal and perinatal risk factors.

**Table 6:** Performance of models using original feature-engineered data.

Model	Acc.	Bal. Acc.	Precision	Recall	F1	AUROC
ET	0.946	0.942	0.953	0.917	0.935	0.993
RF	0.946	0.941	0.963	0.907	0.934	0.993
LGBM	0.945	0.940	0.962	0.907	0.933	0.991
XGB	0.887	0.881	0.890	0.838	0.863	0.952
CB	0.885	0.879	0.880	0.843	0.861	0.945
SVM	0.884	0.877	0.888	0.831	0.858	0.936
GB	0.825	0.818	0.810	0.768	0.789	0.902
LR	0.803	0.797	0.773	0.757	0.765	0.865

ETC achieved the best performance in this setting, with an accuracy of 0.946, balanced accuracy of 0.942, F1-score of 0.935, and AUROC of 0.993. Its strong performance can be explained by its randomized ensemble structure, which improves robustness and captures complex feature interactions. RF achieved similar accuracy but showed slightly lower recall, indicating that some asphyxia cases were still missed. LightGBM also performed competitively because of its ability to model nonlinear decision patterns. In contrast, LR showed weaker performance, likely because its linear decision boundary is less suitable for overlapping clinical risk profiles. GBM also performed lower than the best tree ensembles, possibly due to sensitivity to class distribution and feature noise. These results establish a strong baseline while motivating further experiments with imbalance-aware learning strategies.

### 4.3 Performance with conventional SMOTE-based oversampling

The second experiment was conducted using standard SMOTE to examine whether data-level balancing could improve the recognition of neonatal asphyxia cases. After establishing the baseline performance on the original feature-engineered dataset, this experiment focused on improving minority-class representation by generating synthetic asphyxia samples in the training set. The purpose was to determine whether increasing the availability of minority-class examples could strengthen the learned decision boundary and reduce the tendency of models to favor the majority non-asphyxia class. As reported in Table 7, SMOTE improved the recall and F1-score of several models compared with the original-data setting, indicating better sensitivity toward asphyxia cases. Among the evaluated models, Extra Trees with SMOTE achieved the strongest performance, with an accuracy of 0.951, balanced accuracy of 0.951, recall of 0.951, F1-score of 0.942, AUROC of 0.994, and AUPRC of 0.991. This improvement can be attributed to the ability of Extra Trees to capture nonlinear interactions among perinatal risk factors while benefiting from the additional synthetic minority samples generated by SMOTE. Its randomized tree-splitting mechanism also helps reduce variance and makes the model more robust to feature-level noise.

**Table 7:** Performance of models using standard SMOTE.

Model	Acc.	Bal. Acc.	Precision	Recall	F1	AUROC
ETC	0.951	0.951	0.933	0.951	0.942	0.994
RF	0.949	0.949	0.934	0.948	0.941	0.993
LGBM	0.944	0.939	0.955	0.910	0.932	0.991
CatBoost	0.880	0.878	0.852	0.868	0.860	0.948
XGBoost	0.877	0.875	0.851	0.861	0.856	0.951
SVM	0.873	0.872	0.841	0.863	0.852	0.938
GBM	0.819	0.817	0.778	0.804	0.791	0.903
LR	0.798	0.797	0.745	0.795	0.769	0.865

Random Forest with SMOTE also performed competitively, but its performance remained slightly lower than Extra Trees. This may be because Random Forest selects more optimized split points within bootstrap samples, whereas Extra Trees introduces stronger randomization during tree construction, which can improve generalization when synthetic samples are added to the training distribution. Overall, the SMOTE experiment shows that data-level oversampling improves minority-class learning compared with the baseline setting. However, conventional SMOTE generates synthetic samples through generic interpolation and does not explicitly prioritize difficult or borderline asphyxia cases. This limitation motivates the subsequent evaluation of class-weighted and imbalance-aware models, followed by the proposed HEM-SMOTE approach.

### 4.4 Performance of class-weighted and imbalance-aware models

The third experiment was conducted to evaluate whether algorithm-level imbalance handling could improve neonatal asphyxia detection without modifying the original training distribution through synthetic oversampling. Unlike SMOTE, which balances the data by generating new minority-class samples, class-weighted learning modifies the training objective by assigning a higher penalty to misclassified asphyxia cases. This experiment was therefore designed to examine whether cost-sensitive learning could improve sensitivity toward the clinically important minority class while avoiding the risk of generating less representative synthetic samples. As shown in Table 8, class-weighted and imbalance-aware tree-based models achieved strong predictive performance. Balanced RF and weighted ETC obtained the best results in this experimental group, with an accuracy of 0.952, balanced accuracy of 0.953, recall of 0.958, and F1-score of 0.944. Compared with the standard SMOTE setting, these models provided a further improvement in recall, indicating that algorithm-level imbalance handling was particularly effective in reducing missed asphyxia cases. This is clinically important because false-negative predictions may delay timely intervention for high-risk neonates.

**Table 8:** Performance of class-weighted and imbalance-aware models.

Model	Acc.	Bal. Acc.	Precision	Recall	F1	AUROC
BRF	0.952	0.953	0.931	0.958	0.944	0.993
ETC (W)	0.952	0.953	0.931	0.958	0.944	0.993
RF (W)	0.951	0.951	0.932	0.955	0.943	0.993
LGBM (W)	0.947	0.943	0.958	0.915	0.936	0.992
CatBoost (W)	0.880	0.879	0.846	0.875	0.860	0.948
SVM (W)	0.879	0.876	0.855	0.860	0.857	0.935
XGB (W)	0.878	0.876	0.853	0.861	0.857	0.952
LR (W)	0.798	0.797	0.745	0.795	0.769	0.865

The strong performance of Balanced RF can be attributed to its internal class-balancing mechanism, which trains each tree on balanced bootstrap samples and reduces the dominance of the majority non-asphyxia class. Similarly, weighted ETC benefits from both randomized ensemble learning and class-weighted optimization, allowing the model to capture nonlinear perinatal risk interactions while assigning greater importance to asphyxia-positive samples. Overall, this experiment demonstrates that cost-sensitive and imbalance-aware learning improves minority-class detection more effectively than relying on the original data distribution alone. However, these methods do not enrich the minority-class feature space; they only adjust the learning process to penalize minority-class errors. Therefore, the next experiment evaluates stacking, threshold tuning, and voting ensembles to determine whether model-level generalization and decision-boundary adjustment can further improve predictive robustness beyond individual class-weighted models.

### 4.5 Performance of stacking, threshold tuning, and voting ensembles

The stacking ensemble was developed using XGBoost, LightGBM, and CatBoost as base learners, followed by a class-weighted Random Forest meta-learner. The proposed stacking model achieved an accuracy of 0.947 and an F1-score of 0.937. Threshold tuning improved precision and specificity but reduced recall, indicating that threshold adjustment reduced false positives at the cost of more false negatives. In addition, a voting ensemble was constructed using the best-performing models from previous experiments, namely Balanced Random Forest, weighted Extra Trees, and weighted Random Forest. The hard-voting ensemble matched the performance of the strongest individual class-weighted models but did not further improve the results. The ensemble results are shown in Table 9.

**Table 9:** Performance of stacking, threshold-tuned, and voting ensemble models.

Model	Acc.	Bal. Acc.	Precision	Recall	F1	AUROC
Stacking	0.947	0.944	0.952	0.921	0.937	0.989
Threshold-tuned stacking	0.948	0.942	0.969	0.906	0.936	0.989
Hard voting	0.952	0.953	0.931	0.958	0.944	–
Weighted hard voting	0.952	0.953	0.931	0.958	0.944	–
Soft voting	0.951	0.951	0.932	0.955	0.943	0.993
Weighted soft voting	0.951	0.951	0.932	0.955	0.943	0.993

### 4.6 Performance of the proposed HEM-SMOTE approach

The final experiment evaluated the proposed HEM-SMOTE approach to determine whether hard-example-guided synthetic oversampling could provide a more informative minority-class representation than conventional SMOTE. This experiment was motivated by the limitation observed in previous settings: standard SMOTE improves class balance through generic interpolation, while class-weighted models improve minority-class attention by modifying the learning cost, but do not enrich the minority-class feature space. Therefore, HEM-SMOTE was introduced to generate synthetic asphyxia samples using a hybrid Euclidean–Mahalanobis distance strategy, allowing the oversampling process to consider both local similarity and covariance-aware relationships among perinatal risk factors.

As shown in Table 10, HEM-SMOTE with ETC achieved the strongest overall performance among the HEM-SMOTE-based models, obtaining an accuracy of 0.953, balanced accuracy of 0.953, precision of 0.943, recall of 0.947, specificity of 0.958, F1-score of 0.945, AUROC of 0.994, and AUPRC of 0.992. The strong performance of ETC can be attributed to its randomized ensemble structure, which is effective in capturing nonlinear interactions among maternal and perinatal risk factors while reducing variance. When combined with HEM-SMOTE, the model benefits from a more informative synthetic minority distribution, enabling better discrimination between asphyxia and non-asphyxia cases. we compared with standard SMOTE + Extra Trees, the proposed HEM-SMOTE + Extra Trees improved accuracy from 0.951 to 0.953 and F1-score from 0.942 to 0.945. More importantly, it improved precision and specificity, indicating that the proposed method reduced false-positive predictions while maintaining strong minority-class detection. Although class-weighted models achieved slightly higher recall, HEM-SMOTE + Extra Trees provided a more balanced overall performance by jointly maintaining high recall, specificity, F1-score, AUROC, and AUPRC. This balance is important in neonatal asphyxia prediction because the model should detect high-risk neonates without producing excessive false alarms.

**Table 10:** Performance of the state of the art using the proposed HEM-SMOTE approach.

Model	Acc.	Bal. Acc.	Precision	Recall	F1	AUROC
ETC	0.953	0.953	0.943	0.947	0.945	0.994
BRF	0.952	0.952	0.933	0.955	0.944	0.994
Soft voting	0.952	0.952	0.933	0.955	0.944	0.994
RF	0.951	0.951	0.935	0.952	0.943	0.994
Hard voting	0.951	0.951	0.935	0.952	0.943	–
LGBM	0.947	0.943	0.958	0.915	0.936	0.992
XGB	0.879	0.876	0.859	0.856	0.857	0.950
CB	0.878	0.877	0.848	0.868	0.858	0.946

### 4.7 Repeated validation and statistical analysis

To assess the stability of the principal imbalance-handling strategies, repeated stratified cross-validation was performed using only the training partition. A five-fold stratified cross-validation procedure with three repetitions was adopted, producing 15 paired validation evaluations for each experimental condition. The same folds were used for all compared methods to ensure a consistent paired comparison. The repeated cross-validation results, reported as mean ± standard deviation, are presented in Table 11. Within each fold, missing-value imputation was fitted using only the fold-training subset and then applied to the corresponding validation subset. Standard SMOTE and HEM-SMOTE were applied exclusively to the fold-training data, whereas the validation data remained unchanged. Four Extra Trees configurations were evaluated: no resampling, class-weighted learning, standard SMOTE, and HEM-SMOTE. Accuracy, balanced accuracy, precision, recall, specificity, F1-score, AUROC, and AUPRC were recorded for every validation run. To determine whether the performance difference between HEM-SMOTE and standard SMOTE was stable across the repeated validation partitions, paired differences were calculated for each metric. The mean paired difference, standard deviation, and 95% confidence interval were estimated using the 15 matched evaluations. These results are reported in Table 12. After repeated cross-validation, standard SMOTE and HEM-SMOTE were trained using the complete training partition and evaluated on the same untouched test set of 4,104 cases. The held-out test results are presented in Table 13. Since both methods generated predictions for the same test observations, an exact two-sided McNemar test was performed to compare their paired error patterns. The McNemar contingency table and corresponding *p*-value are reported in Table 14. Statistical significance was assessed at α = 0.05. The detailed interpretation of the repeated cross-validation, held-out evaluation, and McNemar test is provided in Section 4.8.

**Table 11:** Repeated cross-validation results (mean ± standard deviation).

Model	Acc.	Bal. Acc.	Precision	Recall	F1	AUROC
No resampling	**0.9498 ± 0.0029**	0.9456 ± 0.0032	**0.9620 ± 0.0071**	0.9181 ± 0.0081	0.9395 ± 0.0036	**0.9939 ± 0.0006**
Class-weighted	0.9490 ± 0.0036	**0.9487 ± 0.0035**	0.9338 ± 0.0077	**0.9468 ± 0.0055**	**0.9402 ± 0.0041**	**0.9939 ± 0.0006**
Standard SMOTE	0.9479 ± 0.0033	0.9474 ± 0.0032	0.9342 ± 0.0075	0.9438 ± 0.0062	0.9389 ± 0.0038	0.9938 ± 0.0006
HEM-SMOTE	0.9482 ± 0.0036	0.9475 ± 0.0032	0.9358 ± 0.0096	0.9427 ± 0.0061	0.9392 ± 0.0040	0.9938 ± 0.0006

Bold values indicate the numerically best mean performance for each evaluation metric among the compared data-balancing strategies. Tied best values are also shown in bold.

**Table 12:** Paired repeated cross-validation differences between HEM-SMOTE and standard SMOTE.

Metric	Paired evaluations	Mean difference	Difference SD	95% CI
Accuracy	15	0.000284	0.002844	[−0.001291, 0.001859]
Balanced accuracy	15	0.000101	0.002717	[−0.001403, 0.001606]
Precision	15	0.001622	0.007898	[−0.002752, 0.005996]
Recall/sensitivity	15	−0.001102	0.006544	[−0.004726, 0.002522]
Specificity	15	0.001304	0.006444	[−0.002264, 0.004873]
F1-score	15	0.000256	0.003230	[−0.001532, 0.002045]
AUROC	15	0.000015	0.000116	[−0.000049, 0.000080]
AUPRC	15	0.000011	0.000162	[−0.000078, 0.000101]

**Table 13:** Held-out test performance of standard SMOTE and HEM-SMOTE.

Model	Acc.	Bal. Acc.	Precision	Recall	Specificity	F1	TN	FP	FN	TP
Standard SMOTE + ET	0.9493	0.9491	0.9337	**0.9477**	0.9505	0.9407	2,247	117	**91**	**1,649**
HEM-SMOTE + ET	**0.9535**	**0.9526**	**0.9433**	0.9471	**0.9581**	**0.9452**	**2,265**	**99**	92	1,648

Bold values indicate the numerically preferable result in each column. Higher values are preferred for accuracy, balanced accuracy, precision, recall, specificity, F1-score, TN, and TP, whereas lower values are preferred for FP and FN.

Table 14McNemar test for HEM-SMOTE and standard SMOTE.

**Table:** 

	Standard SMOTE	
HEM-SMOTE	Correct	Incorrect
Correct	3871	42
Incorrect	25	166

**Table:** 

McNemar test component	Value
Standard SMOTE correct, HEM-SMOTE incorrect (*b*)	25
HEM-SMOTE correct, Standard SMOTE incorrect (*c*)	42
Total discordant predictions (*b*+*c*)	67
Exact two-sided McNemar *p*-value	0.0498
Significant at α = 0.05	Yes

The exact two-sided McNemar test identified a borderline statistically significant difference in the paired error patterns of the two models (*p* = 0.0498). Because the result is close to the 0.05 threshold, it should be interpreted cautiously.

### 4.8 McNemar test

To determine whether the difference between HEM-SMOTE and standard SMOTE was statistically meaningful on the independent test set, an exact two-sided McNemar test was applied. This test is appropriate because both models were evaluated on the same 4,104 test cases and therefore produced paired predictions for each observation. As shown in Table 14, both methods correctly classified 3,871 cases and jointly misclassified 166 cases. Among the discordant predictions, standard SMOTE correctly classified 25 cases that HEM-SMOTE misclassified, whereas HEM-SMOTE correctly classified 42 cases that standard SMOTE misclassified. The exact McNemar test yielded a *p*-value of 0.0498.

Since the obtained *p*-value was slightly below the significance threshold of 0.05, the paired error patterns of the two methods were considered statistically different on the held-out test set. However, because the result was very close to the threshold, it was interpreted cautiously. The findings indicate a modest held-out advantage for HEM-SMOTE, mainly due to its lower number of false-positive predictions, rather than a large or consistently observed improvement across all validation settings.

### 4.9 Confusion matrix analysis with state-of-the-art methods

**Figure 5:**
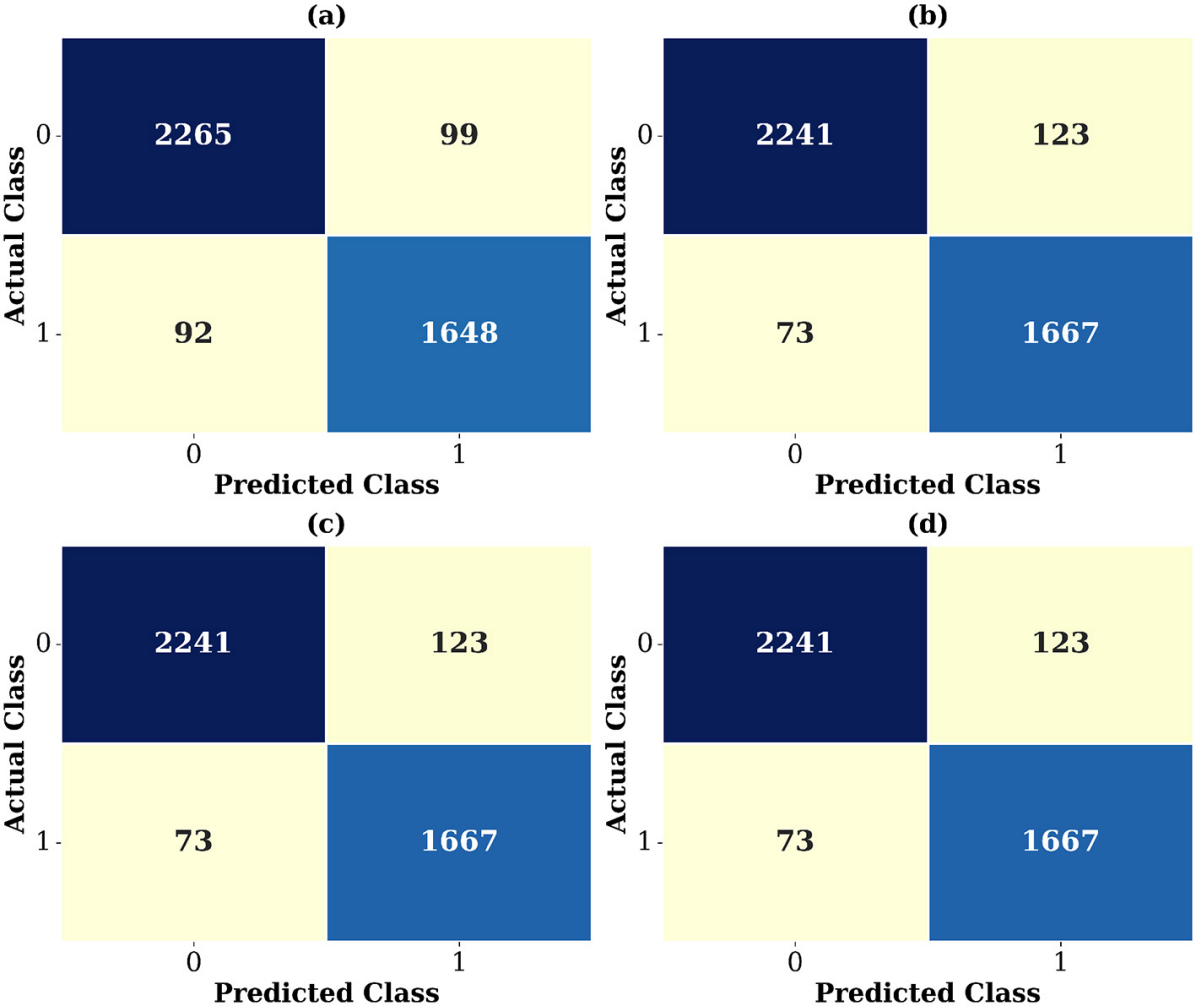
Confusion matrices of the best-performing models for neonatal asphyxia prediction: **(a)** HEM-SMOTE + Extra Trees, **(b)** Balanced Random Forest, **(c)** Weighted Extra Trees, and **(d)** Hard Voting Ensemble.

The confusion matrix results provide further insight into the error distribution of the top-performing models (Figure 5). The proposed HEM-SMOTE + Extra Trees model correctly classified 2,265 non-asphyxia cases and 1,648 asphyxia cases, while producing 99 false positives and 92 false negatives. In comparison, Balanced Random Forest and weighted Extra Trees produced fewer false negatives but more false positives. This indicates that HEM-SMOTE + Extra Trees achieved a stronger balance by reducing false-positive predictions and improving specificity.

**Figure 6:**
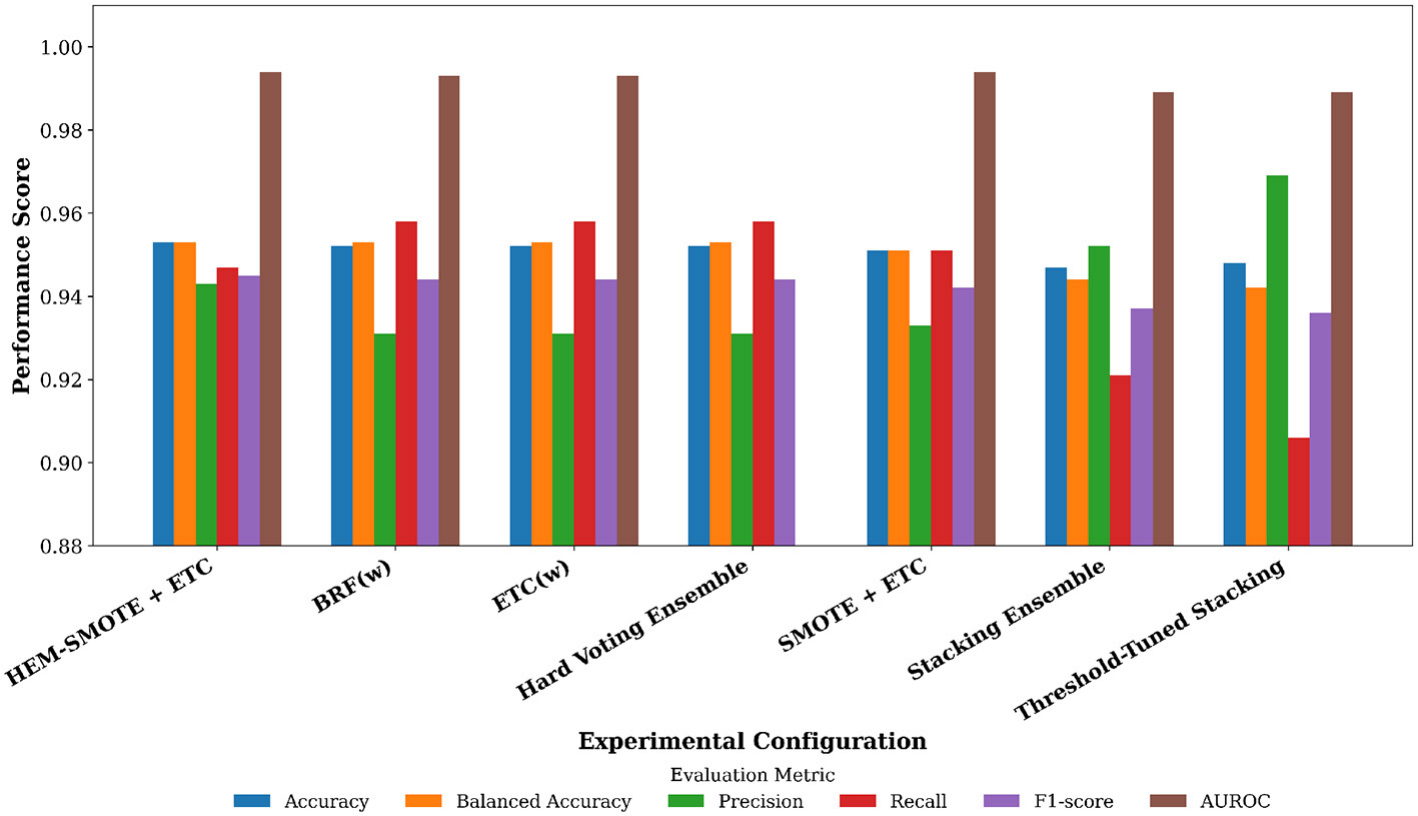
Multi-metric performance comparison of the top-performing experimental configurations for neonatal asphyxia prediction.

Figure 6 compares the top-performing experimental configurations across accuracy, balanced accuracy, precision, recall, F1-score, and AUROC. The proposed HEM-SMOTE + ETC achieved the strongest overall balance, with accuracy and balanced accuracy of 0.953, precision of 0.943, recall of 0.947, F1-score of 0.945, and AUROC of 0.994, indicating stable performance across both majority and minority classes. The class-weighted BRF, class-weighted ETC, and hard voting ensemble produced slightly higher recall values of 0.958, but their lower precision of 0.931 suggests more false-positive predictions compared with the proposed model. SMOTE + ETC also showed competitive performance with an AUROC of 0.994, but its F1-score remained slightly lower than HEM-SMOTE + ETC. In contrast, the stacking-based models achieved higher precision, particularly threshold-tuned stacking with 0.969 precision, but this improvement came with reduced recall of 0.906, indicating a weaker ability to identify asphyxia cases. Overall, the figure shows that HEM-SMOTE + ETC provides the most clinically balanced configuration by maintaining high discrimination, sensitivity, and F1-score simultaneously.

### 4.10 Comparison with recent studies

Recent machine-learning research on neonatal asphyxia and related neonatal-risk prediction have reported variable performance depending on the prediction target, dataset size, feature modality, class distribution, and validation design as shown in Table 15. Therefore, the reported accuracy values should be interpreted within the methodological context of each study rather than as a direct one-to-one benchmark. For example, the study Darsareh et al. (34) reported 99% accuracy for predicting birth asphyxia using RF; however, their dataset contained only 380 asphyxia cases among 8,888 deliveries, indicating a highly imbalanced setting in which accuracy alone may overestimate clinical utility. Similarly, the study Dey et al. (35) achieved very high accuracy using infant cry audio and MFCC-based features with random oversampling, but this modality differs from routinely available structured maternal and perinatal clinical records. The study Onu (36) also used infant cry signals and reported lower accuracy, showing that audio-based approaches may be sensitive to signal quality, recording conditions, and dataset scale. Other studies, such as Tarimo et al. (37), Alhasson et al. (38), and Khatibi et al. (39), focused on predicting low Apgar scores, which are clinically related to neonatal compromise but are not identical to direct neonatal asphyxia classification. Another study Robi and Sitote (40) reported strong performance using a stacking model, but their task involved broader neonatal disease prediction rather than a focused binary asphyxia endpoint. In contrast, the proposed study focuses specifically on binary neonatal asphyxia prediction using structured maternal and perinatal clinical features and introduces HEM-SMOTE, a hybrid Euclidean–Mahalanobis oversampling strategy designed to improve minority-class representation. Although some existing studies reported higher accuracy, the proposed HEM-SMOTE + Extra Trees model provides competitive overall performance while reporting a broader clinical evaluation profile, including precision, recall, specificity, F1-score, AUROC, and AUPRC. Thus, the contribution of this study lies not only in predictive performance but also in the proposed imbalance-aware synthetic sample generation strategy for structured neonatal asphyxia prediction.

**Table 15:** Critical comparison with existing neonatal asphyxia and related neonatal-risk prediction studies.

Study	Prediction target	Best method	Imbalance/strategy	Best performance	Critical limitation/comparative gap
Onu (36)	Perinatal asphyxia detection using infant cry signals	SVM-based pattern recognition	NR	Accuracy up to 88.85%	Used infant cry audio rather than structured maternal and perinatal clinical variables. The performance was lower than the proposed study and was tested as a low-resource diagnostic prototype.
Acan (48)	Outcome prediction of asphyxiated patients using temporal clinical observations	Modified AlexNet with recurrence plots	NR	Average accuracy = 93.3%	Focused on outcome/severity prediction after asphyxia rather than early binary asphyxia prediction. The reported accuracy was lower than the proposed HEM-SMOTE + Extra Trees model.
Mooney et al. (49)	HIE prediction following perinatal asphyxia using clinical variables	Random Forest	NR	Sensitivity = 56–100%; Specificity = 78–99%	Predicts HIE among infants with perinatal asphyxia, not direct asphyxia occurrence. The wide sensitivity range indicates variable model behavior depending on feature combinations.
Darsareh et al. (34)	8,888 deliveries with only 380 birth-asphyxia cases	Random Forest	NR	Accuracy = 99%	Reported very high accuracy, but the positive class was very small, so accuracy alone may overestimate clinical performance. No new synthetic oversampling strategy was proposed.
Alhasson et al. (38)	Low APGAR score prediction using maternal, fetal, and perinatal factors	Random Forest with grid-search CV	NR	Accuracy = 96%; Precision = 98%; Recall = 97%; F1-score = 97%	Strong performance, but the target was low APGAR score rather than direct neonatal asphyxia. The study did not introduce a new data-level imbalance method.
Khatibi et al. (39)	Neonatal 5-minute Apgar score prediction using a national registry dataset	Stacked ensemble	Distributed feature selection and big-data analytics	Accuracy = 99.37%; Precision = 99.37%; Recall = 99.50%; F-score = 99.41%	Very strong performance, but the prediction target was Apgar score category rather than binary neonatal asphyxia. It used a large national registry and is therefore not directly comparable to hospital-level asphyxia prediction.
**Proposed study**	**Binary neonatal asphyxia prediction using structured maternal and perinatal clinical features**	**HEM-SMOTE + Extra Trees**	**Proposed HEM-SMOTE**	**Accuracy = 95.35%;** **Precision = 94.33%;** **Recall = 94.71%;** **Specificity = 95.81%;** **F1-score = 94.52%;** **AUROC = 99.36%;** **AUPRC = 99.15%**	**Provides higher performance than several related lower-accuracy studies and remains competitive with high-accuracy studies while introducing a new hybrid Euclidean–Mahalanobis oversampling strategy for minority-class learning**.

Bold text identifies the proposed study and highlights its prediction target, proposed method, imbalance-handling strategy, performance results, and comparative contribution.

## 5 Conclusion and future work

This study proposed an imbalance-aware machine-learning framework for neonatal asphyxia prediction using feature-engineered maternal and perinatal clinical features. To improve minority-class learning, a hybrid oversampling method, HEM-SMOTE, was proposed by combining Euclidean and Mahalanobis distances for synthetic sample generation. The proposed method was evaluated against original data, standard SMOTE, class-weighted learning, stacking, voting ensemble, and threshold-tuned models. The experimental results showed that tree-based models performed better than linear and kernel-based classifiers. Among all evaluated approaches, HEM-SMOTE with Extra Trees achieved the best overall performance, with 95.35% accuracy, 94.33% precision, 94.71% recall, 95.81% specificity, 94.52% F1-score, 99.36% AUROC, and 99.15% AUPRC. Although class-weighted Balanced Random Forest and weighted Extra Trees achieved slightly higher sensitivity, HEM-SMOTE with Extra Trees provided the strongest overall balance across most evaluation metrics. These findings suggest that hybrid distance-based oversampling can improve minority-class representation and enhance neonatal asphyxia prediction using structured clinical data. This study has some limitations. First, the dataset contains a limited set of maternal and perinatal variables; adding laboratory biomarkers, fetal monitoring signals, and treatment-related information may improve prediction. Second, the current work focuses only on binary classification, while clinical practice may require severity-level or stage-wise risk prediction. Future work will focus on validating the proposed HEM-SMOTE framework using larger and more diverse datasets. Explainable AI methods, such as SHAP-based interpretation, will be explored to improve clinical transparency. Future studies may also extend the framework to multi-class asphyxia severity prediction, temporal modeling of perinatal events, and integration with electronic health record systems for real-time decision support.

## Data Availability

The original contributions presented in the study are included in the article/supplementary material, further inquiries can be directed to the corresponding author.
